# Risk, Reliability and Resilience: Phytolith Evidence for Alternative ‘Neolithization’ Pathways at Kharaneh IV in the Azraq Basin, Jordan

**DOI:** 10.1371/journal.pone.0164081

**Published:** 2016-10-19

**Authors:** Monica N. Ramsey, Lisa A. Maher, Danielle A. Macdonald, Arlene Rosen

**Affiliations:** 1 Department of Anthropology, University of Toronto, Toronto, Ontario, Canada; 2 Department of Anthropology, University of California, Berkeley, California, United States of America; 3 Department of Anthropology, University of Tulsa, Tulsa, Oklahoma, United States of America; 4 Department of Anthropology, University of Texas at Austin, Austin, Texas, United States of America; New York State Museum, UNITED STATES

## Abstract

‘Neolithization’ pathway refers to the development of adaptations that characterized subsequent Neolithic life, sedentary occupations, and agriculture. In the Levant, the origins of these human behaviors are widely argued to have emerged during the Early Epipaleolithic (ca. 23 ka cal BP). Consequently, there has been a pre-occupation with identifying and modeling the dietary shift to cereal and grains during this period, which is considered to have been a key development that facilitated increasing sedentism and, eventually, agriculture. Yet, direct evidence of plant use in the form of macrobotanical remains is extremely limited at Epipaleolithic sites and the expected ‘Neolithization’ pathway has not been robustly demonstrated. However, new direct microbotanical phytolith evidence from the large aggregation site of Kharaneh IV, in the Azraq Basin, suggests that increasingly settled occupation was not the result of wild grass and cereal use, but rather the result of a typical hunter-gatherer balance, based on the use of mostly reliable resources supplemented by some risky resources. Moreover, and illustrating this balance, the direct botanical evidence emphases the importance of the wetlands as an under-recognized reliable plant resource. Significantly, the use of these reliable wetland plant resources at Kharaneh IV represents an unexpected ‘Neolithization’ pathway.

## Introduction

The complex dynamics of people-plant interactions intrinsic to hunter-gatherer adaptations were central to the development of the ‘Neolithic’ lifestyle. Many researchers have characterized Epipaleolithic foragers as the link between hunter-gatherers and the first Neolithic farmers (ca. 11,500–10,500 cal. BP). In particular, the Late Epipaleolithic (Natufian period) (ca. 15,500–11,500 cal. BP) has been the subject of the most intense scrutiny [1–20]. However, more recently, it has been argued that ‘Neolithization’—the transition from mobile foragers to settled farmers, including many presumed associated social changes—began during the Early Epipaleolithic period, perhaps as early as 23,000 cal. year BP [21–25]. The economic and social transformations that marked the emergence of a ‘Neolithic’ way of life included sedentism and agriculture [23]. Understanding the economic and social practices, particularly plant-use practices, that facilitated increasing sedentism at Early-Middle Epipaleolithic sites such as Kharaneh IV (19,830–18,600 cal. year BP [26]), is of the utmost importance. Yet, direct evidence of plant use in the form of well-preserved macrobotanical remains is extremely limited during this important period, with the exception of the remarkable assemblage at the 23 ka year old site of Ohalo II [23, 27]. However, the analysis of this unique macrobotanical evidence has concentrated largely on those plant remains that later became the first domesticates (i.e. wild cereals and grasses) [28–30].

The focus on the first domesticated plants is symptomatic of a broader disciplinary preoccupation with identifying and modeling the dietary shift to cereals and grasses, which is considered a key development in the economic transition from foraging to farming, as outlined in Flannery’s [31, 32] Broad Spectrum Revolution. Consequently, there is a tendency for scholars to view plant-use trends from the Late Epipaleolithic (Natufian) (ca.15,500–11,500 cal. year BP) and more recently, the earlier Epipaleolithic periods (ca. 23,000–15,500 cal. year BP), as part of a clear and successful continuum to agriculture [33, 34], or at least as being teleologically determined. As a result, researchers have not adequately considered the complexity of gathering strategies and the choices that hunter-gatherers faced in the Late Pleistocene [11, 27]. Importantly, not contextualizing the use of these wild ancestors of domestic plants within the broader pattern of wild plant collection potentially obscures alternative and unexpected ‘Neolithization’ pathways. The concept, ‘Neolithization’ pathway, refers to the development of adaptations that characterized subsequent Neolithic life, importantly sedentism and agriculture. Yet, the advent of these developments during the earlier Epipaleolithic did not inevitably lead to a Neolithic lifestyle. As Goring-Morris and Belfer-Cohen argue “developments appear to have been directional only in retrospect” [22].

**Fig 1:**
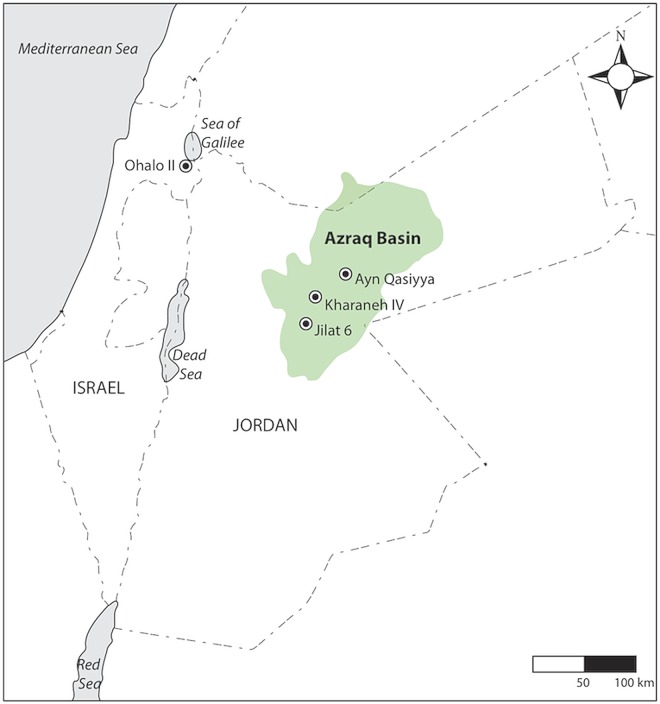
Location map of Kharaneh IV and the other sites mentioned.

The earlier Epipaleolithic could be viewed as a period of ‘Neolithic’ *fits and starts*, whereby some hunter-gatherers transitioned in and out of subsistence and settlement patterns, which later became hallmarks of the Neolithic. This flexible approach is typical of hunter-gatherers and the complex but contextually rational choices foragers’ make when balancing their ecological and cultural environments. In this paper, new direct evidence of plant-use from phytolith analysis conducted on on-site sediments from Kharaneh IV in the Azraq Basin, Jordan (Fig 1), is analyzed to consider how the inhabitants used plant resources. Given there is little local off-site sedimentary evidence to reconstruct the environment through most of the site’s occupation [35], the on-site evidence of plant use is also employed towards reconstructing the local environment. We argue that the evidence demonstrates these people employed a resilient plant use strategy that focused on the selection of risky and reliable resources, which may have facilitated their increasingly sedentary lifestyle and, therefore, represents the origins of an alternative and unexpected ‘Neolithization’ pathway.

This study is the latest from a body of new phytolith evidence attesting to the varied and local nature of Epipaleolithic plant use in the Southern Levant [12, 13, 36–39]. These works join Asouti and Fuller [27] in questioning the idea that Epipaleolithic plant use practices emerged “as ‘pre-adaptations’ *en route* to food production,” and instead view wild cereal and grass use in a manner similar to Savard, Nesbitt and Jones [40], where they form as but one component, within the many local systems of plant use adapted to the different and changing micro-ecologies and historical trajectories’ in the Southern Levant.

The plant use evidence suggests that increasing sedentism, a key component of later ‘Neolithic’ lifestyles, at Kharaneh IV was borne out of a rational balance typical of hunter-gatherer adaptations [41–43], between the use of *risky* resources (i.e. wild cereals, grasses and other seasonal resources) found in the surrounding steppe/parkland landscape and the use of *reliable* resources (i.e. sedges and reeds) found in the wetland landscape, where the site was established. This strategy was *resilient* because by ‘hedging their bets’ on the year-round larder of the wetland, and foraging strategically beyond the safety it afforded, the inhabitants at Kharaneh IV were able to aggregate in large groups and settle for longer than ever before in one place, facilitating the development of a rich social and material existence.

### Late Pleistocene Environment at Kharaneh IV

The Azraq wetland is fed by several springs, which are filled by surface runoff and groundwater [44]. The springs have a low discharge-to-replenishment ratio, with a residence time of 20,000 years [45, 46]. In the past, prior to modern water demands, the springs provided a secure supply of water, even under arid conditions [35, 44, 47]. At present, the much-diminished modern wetland is located approximately 40 km northwest of the site. However, in the past Kharaneh IV was situated on the periphery of the large Pleistocene wetland, surrounded by semi-arid steppe and parkland [35, 44, 48–53]. The wetland would have been a reliable resource-rich environment *relative* to the surrounding steppe and parkland [54], particularly during arid periods. This characteristic of the Azraq Basin has attracted human groups to the region since the Lower Paleolithic [55–58]. These dynamics are not unique to the Azraq Basin and can be seen in other localized wetland contexts in the Levant, including for example the wadi systems in the northwestern Negev Desert dunefields containing Epipaleolithic sites [59]. Similar to the Late Pleistocene water bodies in the Negev Desert dunefield, the high water table characteristic of the Azraq wetland during the Early Epipaleolithic does not necessarily reflect a ‘humid’ phase (i.e. increased precipitation). In the case of the Negev, the development of ancient water bodies was the result of accumulating aeolian sand deposits blocking the wadis, or dune-damming, not increased precipitation [59]. While in Azraq, the aquifers supplying the wetland are fed mainly by groundwater discharge with a millenial scale recharge rate [35], meaning that most of the water accumulating in the Late Pleistocene wetland was a result of increased preciptation thousands of years prior to the LGM.

Recent geoarchaeological analysis by Jones, Maher [35] demonstrates that the wetlands adjacent to Kharaneh IV date to between 23 and 19 ka years ago. At the base of the Early Epipaleolithic occupation (Area B) the wetland deposits are interstratified with the earliest occupation layers. Therefore, it is clear that a rich wetland was located in direct proximity to the site when it was first established. Jones, Maher [35] note that there is little sedimentary evidence from which to reconstruct the environment during occupation of the site. However, they do suggest that the sustained occupation of the site indicates the wetland continued to be a favorable locale for a further 1200 years. Subsequently, the deposition of windblown (loess) deposits and the establishment of an erosional phase between 19 ka and 4 ka BP suggests there was a substantial drying of the wetland and the surrounding landscape [35, 52, 53].

Faunal evidence demonstrates that the inhabitants at Kharaneh IV had available a wide variety of animal resources, including water dependent species such as equids and aurochs, and smaller animals including tortoise and waterfowl [60]. However, they relied most heavily on gazelle (80% NISP in the midden, pit, cache and hearth contexts of area B and 90% in area A) [60, 61]. Based on the skeletal-part profile of the assemblage the authors suggest that hunting occurred close to the site [60]. The consistent long-term hunting practices evident from the faunal assemblage indicates that throughout the sites occupation, the wetland and surrounding steppe and parkland environments were rich in game and provided a dependable supply of animal resources for both food and material manufacture.

### Archaeology at Kharaneh IV

Kharaneh IV (19,830–18,600 cal. BP) [26] is one of the most important Late Pleistocene sites in the Eastern Levant and is one of only two large earlier Epipaleolithic aggregation sites in the Azraq Basin [26, 50, 62]. The other site, Jilat 6, is located 20 km south of Kharaneh IV and is estimated by Garrard and colleagues [50] to be approximately 19,000 m^2^. Notable for its phenomenal size, Kharaneh IV is 21,000 m^2^ with thick archaeological deposits [60, 63]. Radiocarbon evidence shows the site was occupied for 1,225 years [26]. The density of cultural material suggests the site was visited by large numbers of people, staying at the site for long periods of time [26, 60], on a multi-seasonal or possibly year-round basis [64].

Kharaneh IV’s lithic assemblage is significantly different from the assemblage at Jilat 6, while the smaller nearby site of Ayn Qasiyya located in the central Azraq Oasis, features parallels with both lithic assemblages [35, 60, 65, 66]. These varied lithic assemblages suggest that different groups of people from different regions of the Levant converged in the rich wetland environment. These large aggregation settlements may have been established as part of a strategy to claim distinct territories, legitimized through persistent occupation and the construction of socially meaningful places. Supporting this idea, Kharaneh IV features the earliest documented hut structures (of which there are currently three identified) in Jordan [36, 60, 63], a possible subfloor burial, a rich faunal assemblage, worked bone objects, a groundstone assemblage, red ochre and marine shell beads [26, 60, 61, 67]. These material remains provide evidence for increasing sedentism, the use of complex trade networks, sophisticated food processing, personal adornment practices and symbolic behaviors.

**Fig 2:**
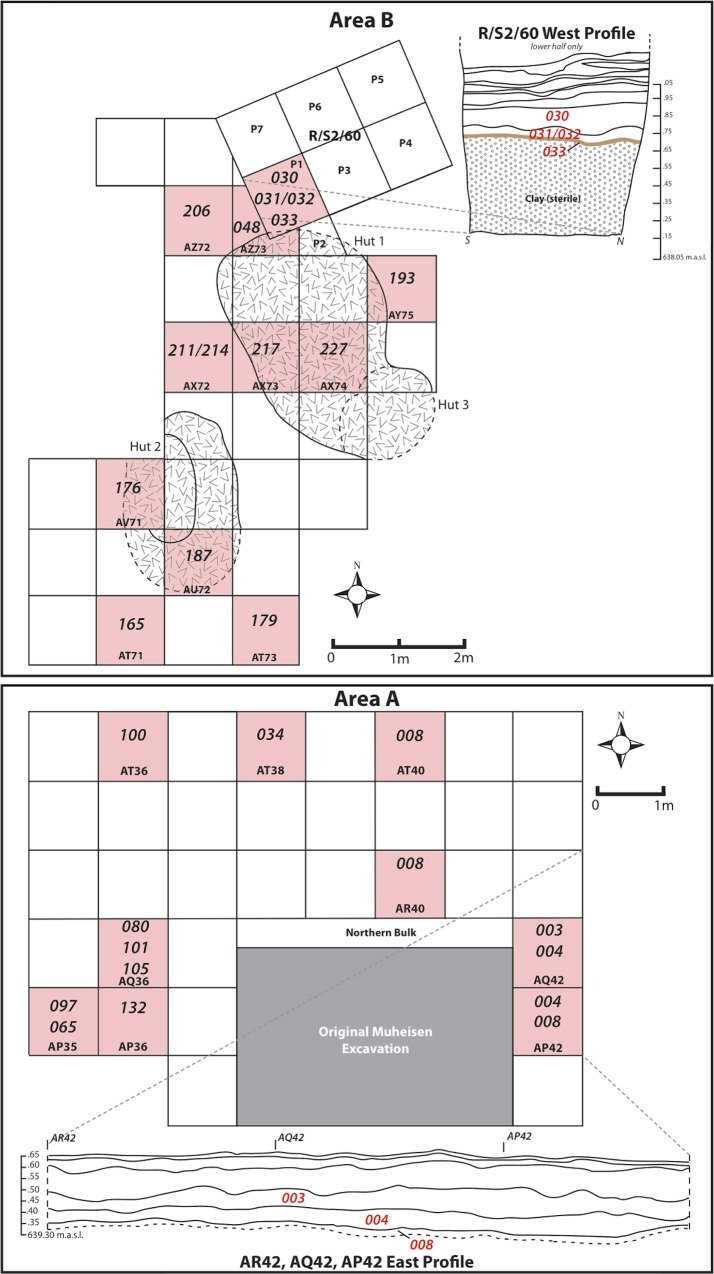
Plan view of key excavation units and profiles at Kharaneh IV. Sampled loci highlighted in red.

Garrard and Price first surveyed the site in the 1970s. Small test excavations were subsequently undertaken in 1981 and 1985 by Muheisen, who excavated three areas totaling ~15 m^2^ (Area A, Area B and a small trench to the north of Area B). Muheisen [68] documented four occupational phases in total, labeled A-D. These phases included two later Geometric Kebaran phases (C and D) and two earlier Kebaran phases (A and B). In 2008 the Epipaleolithic Foragers in Azraq Project (EFAP) renewed excavations at the site [69–72]. EFAP excavations focus mainly, but not exclusively, on two areas of the site, an Early Epipaleolithic occupation (Area B, equivalent to Muheisen’s Kebaran Phase A and B) and a Middle Epipaleolithic occupation (Area A, equivalent to Muheisen’s Geometric Kebaran Phases C and D). Excavation of these occupations provided the samples for this study (Fig 2).

### Modeling the Balance between Risky and Reliable Plant Resource Use at Kharaneh IV

Models of hunter-gatherer adaptation tend to share one basic assumption—that humans are rational decision makers [41–43]. Decision-making can be rooted in social as well as evolutionary ‘reason.’ Yet, in the case of economic decisions (subsistence and settlement), hunter-gatherer scholars have tended to favor one of two explanations for human rationale: either the maximization of optimality, characteristic of optimal foraging theory, or the maximization of fitness, characteristic of human behavioral ecology (HBE) more generally [73]. However, there is growing sentiment supporting the idea that hunter-gatherer decisions were not always driven by maximization goals. Rather, they had only to be good enough to satisfy the range of overlapping interests and needs within the community [73], to ‘satisfice’ rather than to ‘optimize’ [74].

The decision to exploit certain resources is made relative to the other available options. Accordingly, resource selection and ranking decisions were determined at the local level in relation to the unique ecological mosaic (resource aggregation, productivity and predictability) of a region, as well as technological and social factors [75]. Changing climate in the Late Pleistocene would also have greatly impacted the availability of local resources. As such, Rosen and Rivera-Collazo [76] have suggested that Epipaleolithic foragers used an adaptive strategy that cycled in predictable ways from warm/wet phase focus on forest resources to dry/cold phase adaptations which targeted steppic resources–shifting easily back and forth in a cyclical manner from one to another. Recent phytolith work at Ohalo II builds on this model and suggests that wetland resources may also have been particularly important during dry/cold phases [37]. This cyclical adaptation suggests a great resilience, but also important variability in Epipaleolithic responses to these major climatic changes.

In the Southern Levant, phytolith evidence attests to the local nature of hunter-gatherer plant-use practices [38]. Habitats were targeted and the resources selected based on their appeal *relative* to the other options. Consequently, hunter-gatherer lifestyles and the exploitation of specific resources or environments (i.e. wetlands) varied according to the local resource and environment options (wetlands, parkland and steppe). Some earlier Epipaleolithic hunter-gatherer sites provide evidence for a burgeoning Neolithic lifestyle [23]. The unique local ecological factors and historical trajectories of these sites facilitated this transition. The Azraq wetland was an essential part of the ecological setting for Late Pleistocene hunter-gatherers and, therefore, should be a serious consideration when modeling regional plant-use (but see [77]). Indeed, Savard, Nesbitt and Jones’ [40] have suggested that reliable valley bottom plant resources were central to ‘Neolithization’.

Wetlands can provide reliable and perennially available plant resources, specifically aquatic roots, which actually increase in nutrient quality during dry, low-growth periods [78]. However, wetlands should not be construed as lush plant food producing *oases* or *‘Edens’* [79]. Indeed, swamps, marshes, bogs, fens, wet meadows and shallow water are all defined broadly as ‘wetlands’, although each have their own unique characteristics determined mainly by the transition between terrestrial and aquatic habitats [80]. The phytolittoral zone, the vegetated littoral typical of marshes and the edges of some shallow water environments, are identified as the most productive part of the wetlands [80, 81].

As noted by Ramsey and Rosen [38], plants that thrive in this phytolittoral zone include some sedge varieties (*Cyperaceae*), cattails (*Typha* sp.) and reeds (*Phragmites* sp.). All of these plants are of great economic and subsistence value to humans, for the fauna they attract, as well as their own nutritional and favorable ecological qualities [81]. However, compared to other plant resource types, the roots of these aquatic species provide the lowest return rate at ca. 182 kcal/h, compared to terrestrial roots (ca. 2,267 kcal/h), nuts and acorns (ca. 832 kcal/h), and small seeds (ca. 364 kcal/h) ([82], and references therein). Consequently, wetland plant foods might be shown to provide what people need (reliable, but low calories and water), rather than what people want (high calories, variability and flavor) [38].

Steppe environments in the Near East include a variety of economically important wild cereals and grasses, and can be seen to exist between two extremes, forest steppe (parkland) and desert steppe, canopy cover to sparse grass cover [83]. Situated on a transition controlled by precipitation levels, steppe environments can fluctuate greatly in terms of primary productivity. With increased precipitation, forest steppe (parkland) will support fruit, nut and mast ‘orchards’. Importantly, reduced aridity will increase the reliability and length of harvest for these favored resources. Arid steppe environments have a very low primary productivity. In contrast, less arid steppe environments can have relatively high primary productivity [83].

**Fig 3:**
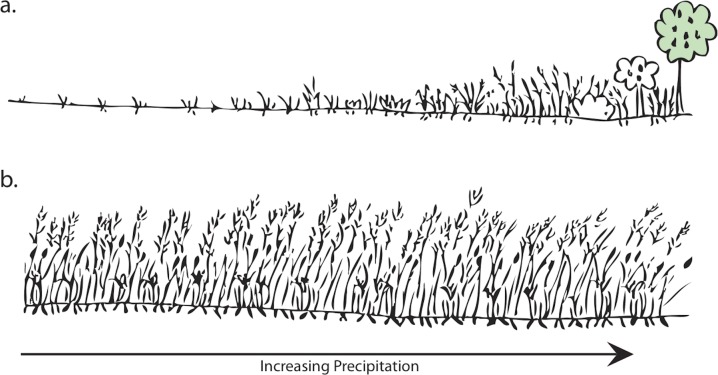
Schematic illustrating the effect of precipitation on a) steppe and b) wetland environments.

The contrasting opportunities found in the wetland versus the steppe/parkland in the Azraq Basin offered hunter-gatherers clear resource options, which forced them to prioritize resources and the levels of risk they were willing to tolerate. While the resource potential of the favored steppe/parkland would have varied, given a sensitivity to changes in climate, the less-favored resources in the wetlands would have provided a reliable fallback (Fig 3). Lee [84] found that the! Kung would eat as much vegetal food as they needed (meeting nutritional needs), but ate as much meat as possible. This pattern is expected according to Lee wherever two or more foods are available. Humans are apt to focus on the more reliable resource (i.e. wetland resources), but still prize the less reliable alternative (i.e. steppe/parkland resources). The push and pull between what people need (predictable foods) and want in their diet (preferable foods) is central to questions about plant-use in the past. This tension means hunter-gatherers do not necessarily choose to remain in the wetland until forced to do otherwise (through exhaustion of resources, social and/or climatic factors).

In regards to climatic factors, it is critical to acknowledge that such trends would have manifest differently in different regions. The southern Levant is characterized by a plethora of micro-habitats. However, archaeologically, we have tended to lump these varied micro-habitats together when considering human adaptation. Indeed, the ‘risky’ environment of the Western Levant during the Last Glacial Maximum may not have been so ‘risky’ in Azraq. Hunter-gatherer decisions are determined at the local level, and accordingly, the way in which climate trends impacted local micro-habitats should be considered.

**Fig 4:**
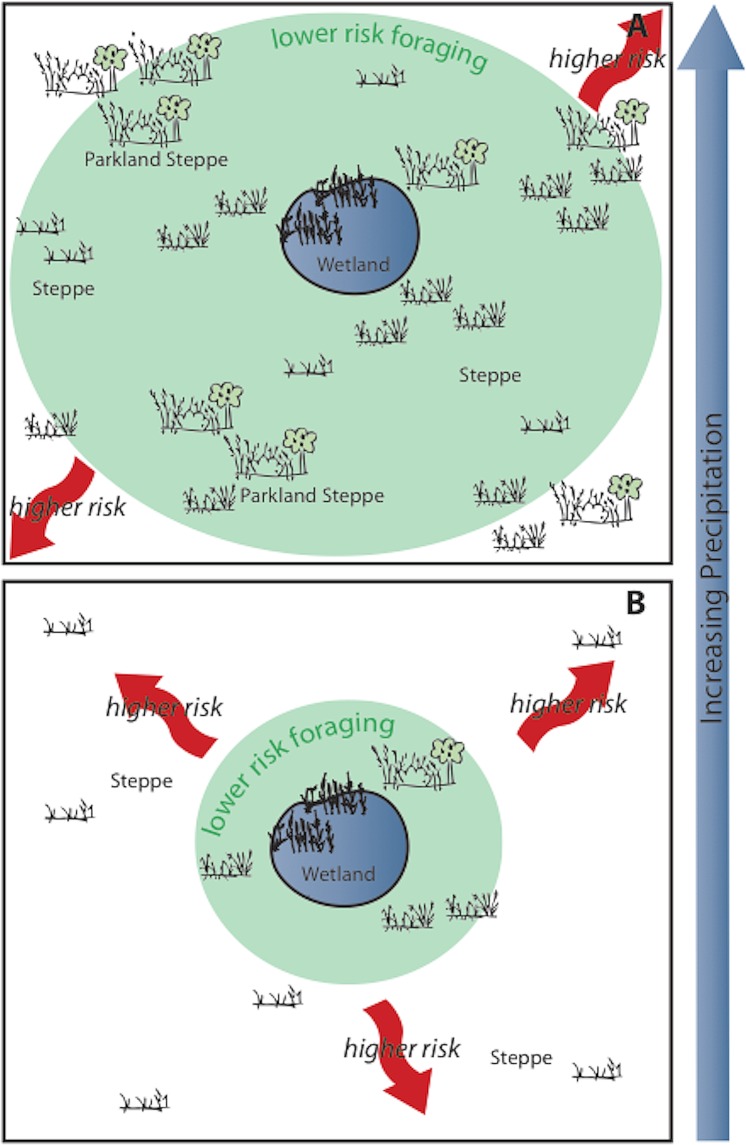
Schematic model illustrating the effect of precipitation on lower risk foraging and higher risk foraging potentials in wetland versus the steppe/parkland zones.

The human preference for preferred foods, a varied diet and, importantly, satisfaction [42, 85], means that during periods of climatic amelioration and increased reliability of the *local* landscape (warm/wet phases), people should have been more willing to take calculated risks, choosing to exploit plant resources outside of the wetland to a greater degree as the region of lower risk foraging expanded (Fig 4). Therefore, during periods of increased aridity and decreased reliability of the surrounding Azraq landscape (cold/dry phases), hunter-gatherers should have relied to a greater extent on the relative productivity and reliability of the wetlands (Fig 4). To paraphrase Bettinger [41]–don’t take chances unless you have to. Wait until the odds are in your favor. This understanding of hunter-gatherer adaptation in combination with direct botanical evidence suggests an alternative ‘Neolithization’ pathway at Kharaneh IV.

## Methods

All necessary permits were obtained for the described study from the Director General, Department of Antiquities of Jordan (Dates: 2008 Ref No. 12-5-2852, 2009 Ref No. 12-5-2404, 2010 Ref No. 12-5-3258), which complied with all relevant regulations. All archaeological phytolith slides produced as part of this study are stored at the University of Toronto, Toronto, Canada, freely accessible upon request from the corresponding author. The modern comparative collection phytolith slides are freely accessible upon request from A. Rosen and are housed in the University of Texas at Austin’s Environmental Archaeology Laboratory. Lastly, the data that informs this paper is available in S1 Table.

### Sediment Sampling

In order to investigate some of the principles outlined above, the senior author analyzed phytolith data from the Epipaleolithic site of Kharaneh IV (Geographical coordinates: 31° 43’ 24 N, 36° 27' 15 E). A total of 31 sediment samples were analyzed for this study (Fig 2, Table 1). The samples were taken from a variety of on-site contexts, including occupation surfaces and features (hearths, dumps and post-holes). Excavations were conducted on a 1 x 1 m grid. However, these squares were further divided into 50 x 50 cm quads or 25 x 25 cm subquads when finer control was needed. The site was excavated following natural and cultural layers, which were further subdivided into 5–10 cm arbitrary levels. All of the excavated sediment was subject to flotation with additional samples collected for micro-artifacts, micro-fauna, micromorphology, and sediment analysis [71]. The phytolith samples for this analysis were collected from the sediment samples taken during the 2008, 2009 and 2010 excavation seasons.

**Table 1:** Sample list with loci descriptions, contexts and lab numbers.

**Area B**	**Loci**	**Description**	**Sample Context**	**Lab Number**
Occupation Surfaces	033	Base of occupation. Light brown lacustrine clay sediments, indicating significantly moister conditions during the initial human occupation. Lithic, faunal and charcoal remains in deposit.	R/S2/60.P1.52	1B.14.1
	032	Part of a series of alternating ‘occupation surfaces’ and refuse. Thin 1–2 cm clay deposit. Dark yellowish brown sediments with a chalky appearance.	R/S2/60.P1.51	1B.13.1
			R/S2/60.P1.51	1B.12.1
	030	Part of a series of alternating ‘occupation surfaces’ and refuse. Yellowish brown silty clay. High number of finds and their jumbled up nature suggests a refuse area.	R/S2/60.P1.46	1B.11.1
	227	Compact yellow/brown layer	AX74.24	1B.18.1
	217	Occupational surface, grayish brown, compact clay.	AX73.21	1B.17.1
Feature (dumps)	165	Loosely compacted bone-dump deposit. Brown, silty sand.	AT71.6	1B.15.1
	179	A mottled deposit with bits of lighter clay and spots of orange-brown material. Artifacts were not very frequent except for seven large, special finds (stone, flint and five large bones including a horn core).	AT73.13	1B.2.1
	206	Dark loose, silty deposit. Few pieces of burnt bone, charcoal, noticeably darker spot isolated within 043. Possibly a dump.	AZ72.11	1B.5.1
	211/214	Dark brown organic rich sediment. Loamy clay. Loose compaction (large hearth or dump).	AX72.23	1B.6.1
	193	Sandy loosely compacted pit fill.	AY75.2	1B.4.1
Feature (pit fill)	176	Compact grey pit fill.	AV71.15	1B.16.1
	187	Sediment associated with fox skull. Light brown soil with clay inclusions.	AU72.16	1B.3.1
**Area A**	**Loci**	**Description**	**Sample Context**	**Lab Number**
Occupation Surfaces	004	Compact brown sediments.	AP42.9C	1A.9.2
			AQ42.14A	1A.26.1
			AQ42.14B	1A.27.1
	008	Compact light brown sediments.	AT40.110	1A.10.1
			AR40.12A	1A.29.1
			AR40.12C	1A.30.1
			AP42.12A	1A.31.1
			AP42.12C	1A.32.1
	100	Compact mottled undulating deposit with high concentrations of charcoal (same as 008, under 034).	AT36.9	1A.15.2
	132	Dark brown sediments	AP36.54	1A.18.1
	003	Dark brown sediment with lots of charcoal	AQ42.10	1A.25.1
	080	Compact sediment with lots of charcoal, flat lying artifacts, bone and shell beads (beneath 100).	AQ36.47	1A.13.1
Feature (hearth)	034	Loamy sand, compact soil, with bones and bits of charcoal. Large ashy feature (beneath 003).	AT38.10	1A.11.1
	065	Dark brown stain, overlapping hearth deposit (067).	AP35.20	1A.12.1
	101	Loose brown sediment patch near hearth (065).	AQ36.17	1A.16.1
Feature (post-hole)	097	Dark brown sediment patch.	AP35.13	1A.14.1
	105	Dark brown sediment patch.	AQ36.16	1A.17.1

### Laboratory Methods

Phytoliths were extracted from the sediments following Rosen’s [86, 87] protocol, which employs a series of techniques to remove carbonates, clays and organics, before extracting the phytoliths. First, the sediment was sieved though a 0.25 mm mesh to remove the coarse sediment fraction. A sub-sample of approximately 800 mg was weighed and taken for analysis. The sample was treated with 30 ml of 10% HCI to remove the carbonates. To disperse the clays, a solution of sodium hexametaphosphate (lab grade Calgon and distilled water) was added to the sample. The clays were removed from the sample by decanting, after settling the fine sands and silts in an eight cm column of water for one hour. This process was repeated until the suspense was clear. Organic matter was removed by dry ashing the samples in a muffle furnace for 2 hours at 500°C. The phytoliths were then extracted from the remaining fraction using heavy density separation. A sodium polytungstate (SPT) solution (with distilled water) calibrated to 2.3 specific gravity was used to separate the phytoliths from the heavier minerals. The phytoliths were then poured off into a clean centrifuge tube, washed in distilled water, dried, weighed and then mounted on microscope slides in Entellan. The phytolith slides were counted at 400x magnification using a transmitted-light microscope (Nikon Eclipse E200). A minimum of 300 single-cells and 50 multi-cells (whenever possible) were counted on each slide. The results are expressed as number per gram of sediment. The absolute counts (number per gm sediment) for each phytolith type were calculated using a modified method outlined by Albert, Lavi [88]; Albert, Weiner [89] see Power, Rosen [90] for details.

### Phytolith Analysis

Phytoliths are microscopic silt-sized particles of opaline silica. They form when plants take up soluble silica from the ground water. The silica is then deposited in and around the intracellular and extracellular spaces, creating durable inorganic silica ‘casts’ of the plants’ cells. This process is genetically and environmentally determined [91, 92]. Grasses, sedges and palms (monocotyledons) readily produce phytoliths, often distinctive to plant family, genus and more rarely, species. Woody trees and other herbaceous dicots also produce phytoliths, although far fewer and with more irregular forms [93]. Indeed, grasses produce 20 times more phytoliths than dicot wood and 16 times more than dicot leaves [94].

**Fig 5:**
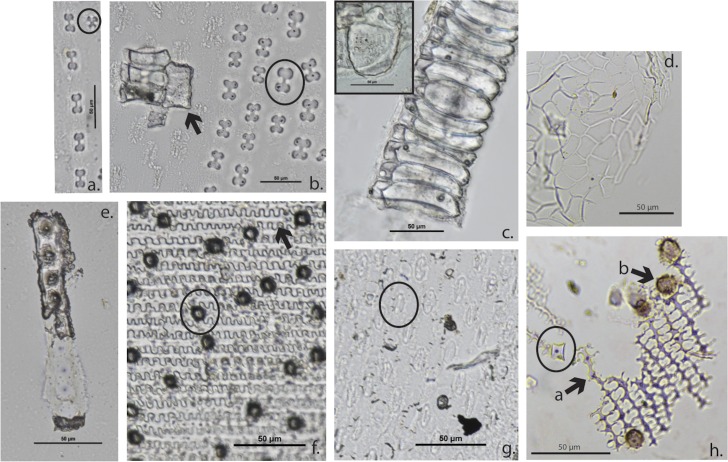
Modern phytolith microfossil comparative examples (scale 50 μm). a. *Arundo donax* (leaf), cross short cell (circled); b. *A*. *donax* (leaf), bulliform (arrow), bilobe short cell (circled); c. *Phragmites australis* (leaf), stacked keystone bulliforms (inset, single cell in plan view); d. *Quercus pubesence* (leaf), Polyhedrons (multi-cell); e. *Cyperus rotundus* (leaf), sedge cones; f. *P*. *australis* (culm), narrow ‘pinched’ short cell (circled), echinate long cell (arrow); g. *P*. *australis* (leaf), ‘hamburger’ stoma (circled); h. *Hordeum spontaneum* (husk), rondel short cell (circled), dendritic long cell (arrow a), papillae (arrow b).

In grasses and other monocots, silica is deposited passively and actively in the cells of the plant. Therefore, phytoliths can form in individual cells, producing single-cell phytoliths, or as a suite of attached adjacent cells producing multi-cell forms, known also as silica skeletons. By studying the anatomical morphology of these fossilized sections of plant tissue it is possible to make identifications down to the plant genus or species level. Single-cell monocotyledon phytoliths are identified according to the ICPN classification system where possible [95]. Key phytolith microfossils employed in this study were identified by the criteria described in Table 2. Modern comparative examples are pictured in Fig 5.

**Table 2:** Phytolith microfossil identification criteria and reference.

Phytolith Morphologies (single-cell unless described as multi-cell)	ICPN alternative	References to identification criteria/comments
Psilate long-cell^**G~**^	Elongate psilate margin	Most frequently found in grass stems [96, 97].
Echinate long-cell^**M**^	Elongate echinate	General of monocots. Of particular importance as a morphology that is found in *Phragmites* (reed) culms (Fig 5F)
Dendritic long-cell^**G**^	Elongate dendritic	Found primarily in pooid grass husks and are characterized by finely branched processes [98, 99]. See the ICPN schematic drawings [95]. (Fig 5H).
Bilobe short-cell^**G**^		Generally panicoid grasses [100] (Fig 5B).
Polylobate short-cell^**G**^		Generally panicoid grasses [100] (Fig 6B).
Cross short-cell^**G**^	Quadralobate	Generally panicoid grasses [100] (Fig 5A).
Saddle short-cell^**G**^		Generally chloridoid grasses [100], but also appears in *Phragmites*.
Rondel short-cell^**G**^		Generally pooid grasses [100] (Fig 5H).
Wild grass husk ^**G**^ **(multi-cell)**		Generally pooid grass. Dendritic long cells, with papillae and short cells (mainly rondel). Cork cells are sometimes silicified (for a more detailed discussion of husk identification methods please refer to [99]) (Fig 5H; Fig 6A).
Bulliform^**G**^		Found in the leaves of grasses, also known as motor-cells [96]. (Fig 5B).
Stacked Bulliforms^**G**^ **(multi-cell)**		Found in the leaves of grasses. Higher silicification may indicate a wet or submerged growing environment [101–103]. (Fig 5B).
Keystone Bulliform (‘Fan-shaped’) (cf. reeds)^**G**^	Cuneiform bulliform cell	Commonly occur in reed-grass species that favor watery habitats [103]. Cf. to fan-shaped reed [92]. With higher silicification may also become a ‘stacked’ multi-cell form (Fig 5C).
*Phragmites* (reed) culm^**G**^ **(multi-cell)**		Echiniate long cells connected by narrow ‘pinched’ short-cells (mainly rondel to saddle). The short-cells are narrower than the echinate long-cells that connect them [104, 105] (Fig 5F; Fig 6D).
*Phragmites* (reed) leaf^**G**^ **(multi-cell)**		Characterized by small frequent stomata [104, 105], with a central lacuna that pinches out beyond the more silicified top and bottom (‘hamburger’ shape) [106] (Fig 5G).
Sedge cones^**M**^		See [107–110] (Fig 5E; Fig 6E). Single and multi-cell forms.
Juncus-type^**M**^		See (Fig 108 and 114A in [106]). Characterized by small, linear stacks of uniform oval to cube shaped cells.
Platelets (sheet)^**D**^^**M**^		See [111]. Found in dicot leaves and wood, cf. to platelet [89].
Polyhedron^**D**^		Found mainly in dicot leaves, single and multi-cell forms [89, 112] (Fig 5D).
Scalloped^**D**^^**M**^		Found mainly in dicot leaves [112] (Fig 6C).
Honeycomb^**D**^^**M**^	Favose	Found mainly in dicot leaves [89, 111].
Tracheids^**D**^^**M**^		Found mainly in dicot leaves, cf. to tracheary [89].
Smooth spheroid ^**D**^		Found mainly in dicot wood, cf. to spheroid psilate [89].
Blocks^**D**^^**M**^		Found mainly in dicot wood, cf. to parallelepiped block forms [89].

Key

^**G**^ grasses

^**G~**^ mainly grasses

^**M**^ monocot

^**D**^ dicot.

## Results and Discussion

**Fig 6:**
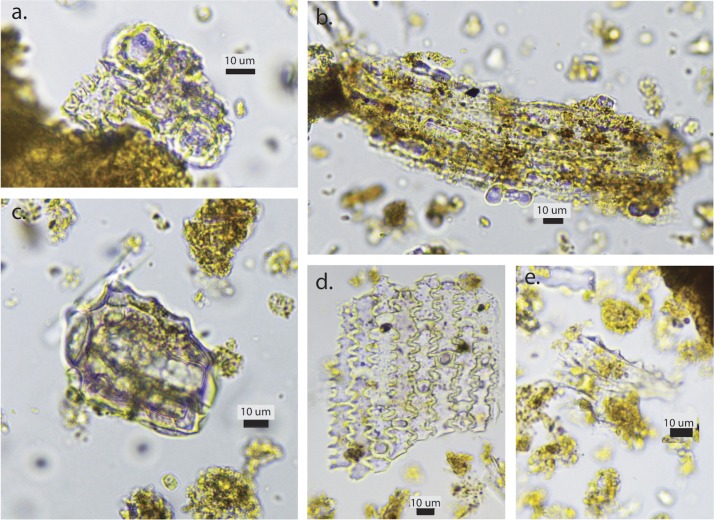
Phytoliths from Kharaneh IV. a. wild grass husk; b. panicoid grass; c. scalloped dicot leaf; d. *Phragmites* sp. culm; e. sedge cones.

The phytoliths throughout the site appear to be well preserved with the presence of delicate morphotypes such as hairs and some large multi-cells, suggesting favorable preservation conditions. Two common monocot phytolith types, ‘psilate long cells’ and ‘rondel’-shaped short cells, dominate all of the samples. Psilate long cells are found in all grasses and sedges and they have limited diagnostic utility, except as indicators of stems (also described as culms) rather than inflorescences, which, are indicated by dendritic long cells (primarily in pooid grasses). However, the dominance of ‘rondel’ short cells is important for the reconstruction of local environmental conditions (Fig 6).

### Vegetation Trends

**Fig 7:**
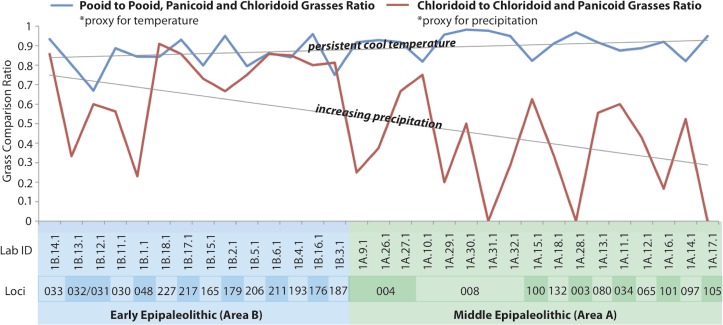
Histogram of Grass Short-cell Comparison Ratios. Pooid to pooid, panicoid and chloridoid grass ratio is a proxy for temperature (higher ratio indicates cooler conditions). Chloridoid to chloridoid and panicoid grass ratio is a proxy for precipitation (higher ratio indicates drier conditions).

The grass sub-families of pooids (with ‘rondel’ morphotypes) are formed in C_3_ grasses and their prevalence indicates a prevailing cool or temperate climate. Chloridoids (indicated by ‘saddle’ short cells) generally form in C_4_ grasses, and panicoids (indicated by ‘cross,’ ‘biolobe’ and ‘polylobe’ short cells) generally form in C_4_ grasses. Relative ratios of these sub-families are employed as a general proxy for temperature and level of aridity (Fig 7). High ratios of pooid to total pooid, chloridoid and panicoid grasses support the interpretation that inhabitants of Early Epipaleolithic Kharaneh IV were exploiting plants from the cooler more temperate micro-habitats that we expect were more prevalent around the site during the LGM. Given chloridoid grasses tolerate arid conditions better than panicoid grasses, the ratio of chloridoid to chloridoid and panicoid grasses is employed as a proxy for aridity, with higher ratios indicating plants from micro-habitats which were more arid, and lower ratios indicating plants from less arid zones [97, 113]. At Kharaneh IV, there is a clear shift from a higher chloridoid ratio to a lower chloridoid ratio from the Early to Middle Epipaleolithic (Fig 7). The evidence suggests that inhabitants of Kharaneh IV were exploiting plants from less arid zones in the steppe and parkland surrounding Kharaneh IV during the Middle Epipaleolithic occupation. This is in keeping with warming and wetting trends, which prevailed in the Southern Levant after the LGM. The finding is important because the primary productivity of steppe environments can fluctuate greatly based on the level of precipitation (Fig 3).

It is possible this shift in vegetation reflects the increase in rainfall during the Middle Epipaleolithic, which resulted in the development of a more productive steppe/parkland in Azraq. This increase in the productive potential of the steppe/parkland should result in the expansion of lower-risk foraging into the wider steppe. A shift in foraging strategy should be evident in the phytolith assemblage through increasing use of steppe/parkland and woodland plant resources during the more humid Middle Epipaleolithic (warm/wet phase) when compared to the comparatively arid Early Epipaleolithic (cold/dry phase).

While human adaptation and plant use is constrained by plant availability, and therefore reflects environmental opportunities, given the assemblages are from on-site contexts, their composition is determined by human behavior. Yet, other on-site botanical assemblages, including the wood charcoal assemblage at El-Wad Terrace in Israel [114] have been employed effectively to reconstruct off-site vegetation. Moreover, as previously noted, at Kharaneh IV contemporaneous off-site sediments are rare and, so far, the on-site contexts provide our only avenue for reconstructing the local vegetation.

### The Balance Between Risky and Reliable Plant Resource Use at Kharaneh IV

**Fig 8:**
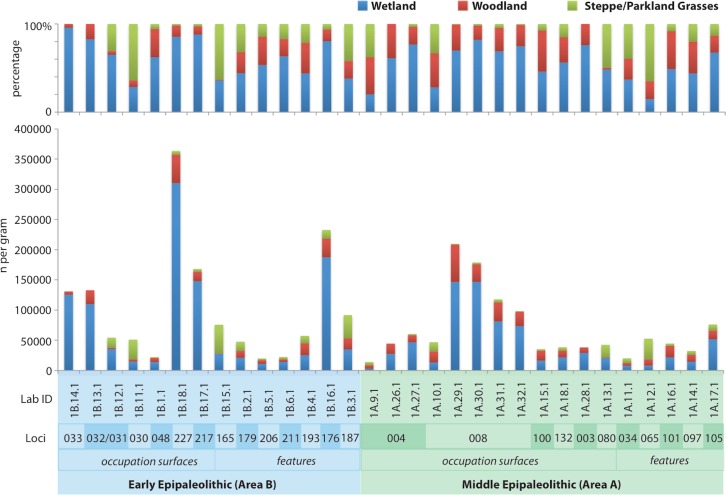
Histogram of ecozone-type phytoliths.

To develop a broad understanding of the foraging strategy employed at Kharaneh IV, the phytoliths are grouped to identify wetland, steppe/parkland grasses and woodland ecozone-types (Table 3, Fig 8). These categories do not necessarily conform to traditional vegetation zones, but rather provide a general picture of plant-use categories. See Ramsey and Rosen [38] and Ramsey, Rosen [37] for a full discussion concerning the use of these categories.

**Table 3:** Phytolith microfossils categorized according to ecozone-type.

Ecozone-type	Phytolith Microfossils
Wetland	Cyperaceae ‘cones’, ‘fan-shaped bulliforms’ (cf. reeds), *Phragmites* sp. culm and leaf, Juncas-type
Woodland	Platelets, honeycomb, scalloped, polyhedron, tracheids, smooth spheroid, blocks, all indet dicots
Steppe/Parkland Grasses	Dendritic long-cells, papillae, all husk multi-cells

From the distribution of ecozone-type phytoliths it is clear that wetland-type resources predominate. However, while phytolith evidence shows that wetland plant resources (reeds and sedges) were employed extensively, without starch evidence from the edible plant parts (seeds or roots) or evidence from contexts that point to consumption, such as groundstone residues, it is not possible at this time to identify how Early and Middle Epipaleolithic peoples at Kharaneh IV employed these resources in their diet. To rectify this research gap more refined context-specific microbotanical analysis and starch analysis of ground stone samples from Kharaneh IV is on-going. In spite of a lack of direct evidence, indirect evidence including ethnographic data from hunter-gatherers subsisting in analogous environments [115–123] and experimental evidence demonstrating the nutritional potential of processed sea club-rush roots (*Bolboschoenus maritiumus* (L.) Palla.) [124–127], a wetland sedge that has been recovered from ancient sites in the Levant and Anatolia [54], points to the importance and reliability of wetland plant-use in the region. Consequently, the prevalence of low-risk wetland-type resources throughout the site’s use demonstrates that the inhabitants of Kharaneh IV employed a risk adverse wetland-oriented adaptation. This is not unexpected [38] and fits with the model of risky and reliable hunter-gatherer resource use described above.

**Fig 9:**
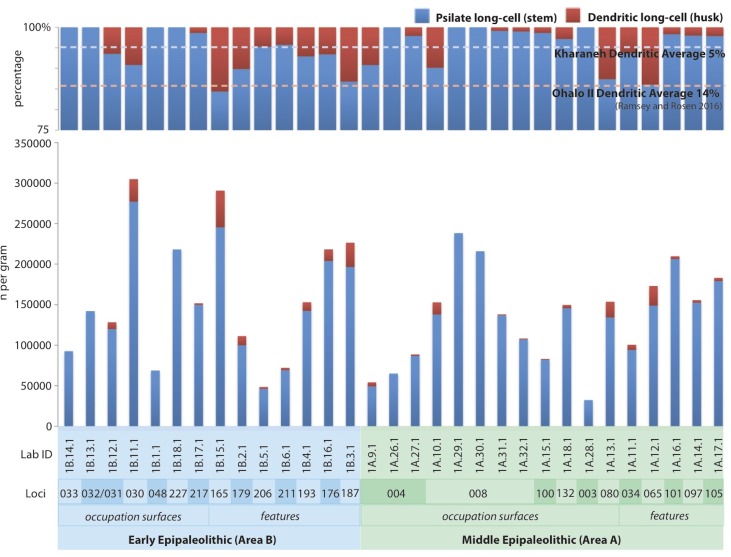
Histogram of psilate (stem) and dendritic (husk) single-cell phytoliths. Percentage scale is 75–100%.

During the Early and Middle Epipaleolithic occupations, steppe/parkland grasses-type resources were also regularly exploited. This suggests that the inhabitants of Kharaneh IV were willing to tolerate higher risk foraging when supported by the reliable resources from the wetland. Based on the presence of dendritic long-cells (grass husks) (Fig 9) it appears that husks were prevalent on some occupational surfaces (loci 030 and 080), but were concentrated particularly in the feature contexts. In the Early Epipaleolithic contexts the features include dumps and pit fills and in the Middle Epipaleolithic contexts the features of interest are hearths (loci 080, 034, 065). The specialized nature of these contexts suggests that the husk remains are the result of wild grass and cereal processing refuse and/or disposal practices.

Comparing psilate long-cells to dendritic long-cells provides an indication of the level of grass and cereal use at the site (Fig 9). It appears that wild grasses and cereals were employed to a lesser degree at Kharaneh IV (5%) than at Ohalo II (14%). This trend noted by Ramsey and Rosen [38] is attributed to the lower levels of risk associated with foraging in the mosaic parkland landscape surrounding Ohalo II. Yet, at Kharaneh IV when increasing productivity in the steppe/parkland may have expanded the lower risk foraging options during the Middle Epipaleolithic occupation, the hunter-gatherers choose not to increase their exploitation of steppe/parkland-type grasses. Rather, steppe/parkland grasses-type resource exploitation remained largely unchanged, while the level of woodland-type resource exploitation increased (Fig 8). This trend towards woodland resource use with the expansion of lower risk foraging, the results of a more humid Middle Epipaleolithic climate, is in keeping with the Epipaleolithic adaptive cycles proposed by Rosen and Rivera-Collazo [76].

**Fig 10:**
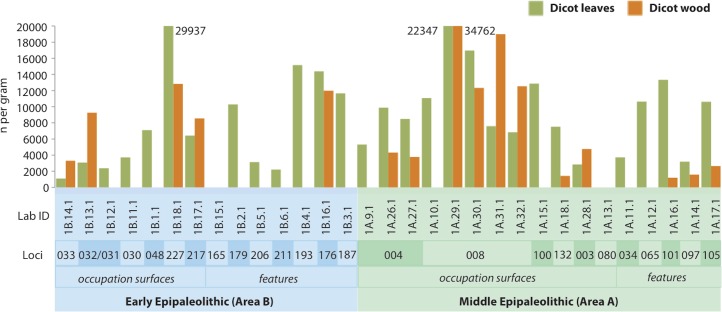
Histogram of dicot leaves and dicot wood phytoliths.

Reviewing the dicot leaf and wood trends (Fig 10), it appears that the inhabitants at Kharaneh IV adjusted their foraging strategy in the Middle Epipaleolithic to include more dicot resources. Considering there is no evidence for a change in wetland-type plant use trends (Fig 8) (i.e. shift from wetland to steppe resources–push factors), it is possible that this shift was facilitated by the increasing productivity of the surrounding parkland (pull factors), but it is unclear if this trend reflects increasing use of dicot resources for fuel, food or material purposes. The use of dicot resources as fuel is supported by the increase of dicot wood. Yet, this increase may also reflect a more general increase in dicot resources for fuel, materials and food. On-going starch analysis and continued investigation of on-site phytolith remains in conjunction with an expanded dicot reference collection will help clarify this trend.

It is important to understand why dicot resources and not wild grass and cereal resources, were prioritized with the possible expansion of lower risk foraging opportunities during the Middle Epipaleolithic occupation of the site. Like Kharaneh IV, Ohalo II also features a wetland-oriented lifestyle [37]. However, it is clear from both the microbotanical (phytolith and starch) [37, 128, 129] and macrobotanical (for example see [130–132]) assemblages at Ohalo II that wild cereals and grasses were an important component of the diet. One explanation for the more muted use of grass resources at Kharaneh IV might be related to the types of grass resources available. It is possible that the rich mosaic environment near Ohalo II on the Sea of Galilee hosted a more preferable variety of grasses, including wild cereals, than the expansive steppe and parkland in the Azraq Basin. Starch and macrobotanical analysis may provide clarification. Even with the expansion of lower risk forging opportunities, the inhabitants at Kharaneh IV may have had no interest in gathering more grass resources.

If hunter-gatherer decisions had only to be good enough to satisfy the range of overlapping interests and needs within the community [73], it is possible that the reliable supply of plant resources in the wetland and the rich assortment of game, meant the inhabitants at Kharaneh IV achieved a sustainable balance between the use of risky and reliable resources, which suited their needs. While the changing landscape may have expanded the lower risk foraging opportunities and potentially facilitated an increase in the use of dicot resources, plant-use strategies remained largely unchanged. Indeed, the contrasting opportunities found in the wetland versus the steppe/parkland in the Azraq Basin offered hunter-gatherers clear options about what plant resources they wanted to prioritize and what levels of risk they were willing to tolerate, and they appear to have chosen to limit their risk by consistently focusing on lower risk foraging opportunities.

## Conclusions: Alternative ‘Neolithization’ Pathways

‘Neolithization’ pathway refers to the development of adaptations that characterized subsequent Neolithic life, sedentary occupations and agriculture. However, the appearance of these incipient behaviors during the earlier Epipaleolithic did not inevitably lead towards a Neolithic lifestyle. Rather, the earlier Epipaleolithic could be viewed as a period of ‘Neolithic’ *fits and starts*. In this paper, we have presented new direct botanical evidence of one such *fit and start*. The evidence from Kharaneh IV has been employed to consider how the inhabitants used plant resources and how their selection of risky and reliable resources resulted in a resilient plant-use strategy that may have facilitated their increasingly sedentary lifestyle, and for that reason, represents the origins of an alternative and unexpected ‘Neolithization’ pathway.

At Kharaneh IV, their increasingly settled lifestyle, a key component of later ‘Neolithic’ lifestyles, is shown in this paper, based on plant use evidence, to have been born out of a rational balance, typical of hunter-gatherer adaptations [41–43], between the use of *risky* resources (i.e. wild cereals, grasses and other seasonal resources) found in the surrounding steppe/parkland landscape, and the use of *reliable* resources (i.e. sedges and reeds) found in the wetland landscape beside which the site itself was established. This strategy was *resilient* because by ‘hedging their bets’ on the year-round larder of the wetland and foraging strategically beyond the safety that afforded, the inhabitants at Kharaneh IV were able to aggregate in large groups and settle for longer than ever before in one place.

These new findings support Asouti and Fuller [27] in questioning the idea that Epipaleolithic plant-use practices emerged as ‘pre-adaptations’ to food production and, importantly, lends support to earlier works that have questioned the importance of grasses and wild cereal resources in the development of sedentism [40, 133]. Indeed, the increasingly intensive settlement seen at Kharaneh IV, based on the phytolith evidence, was not the result of increasing grass and wild cereal use at all, but rather the result of a typical hunter-gatherer balance, based on the use of mostly reliable resources supplemented by some risky resources. In providing direct botanical evidence illustrating this balance, we have also brought to light an under-recognized reliable plant source, the wetland. For this reason, the use of reliable wetland plant resources at Kharaneh IV represents an unexpected ‘Neolithization’ pathway. Importantly, this reinforces Savard, Nesbitt and Jones’ [40] contention that reliable resources were central to ‘Neolithization’.

While, in hindsight, the lifestyle exhibited at Kharaneh IV may represent an incipient step towards ‘Neolithization’, ultimately it was a result of the resilient wetland-oriented hunter-gatherer lifestyle employed by Early and Middle Epipaleolithic peoples. More on-site phytolith analysis and starch analysis in appropriate contexts (focused on features and ground stone tools) is currently underway to further refine how wetland and steppe/parkland starchy resources may have been employed in the diet. These types of analyses should be extended to later-period sites in the Azraq Basin, as we expect the use of reliable wetland plant resources continued to be central to the development of Neolithic life ways and perhaps even facilitated the adoption of cereal cultivation, and eventually agriculture.

## Supporting Information

S1 Table Phytolith Data. Values represent number of phytoliths per gram of sediment (n per gm).
(XLSX)

## References

[1] Anderson P. Harvesting of wild cereals during the Natufian as seen from the experimental cultivation and harvest of wild einkorn wheat and microwear analysis of stone tools In: Bar-Yosef O, Valla FR, editors. The Natufian Culture in the Levant. Ann Arbor: International Monographs in Prehistory; 1991 p. 521–56.

[2] Bar-Yosef O. The Natufian Culture in the Levant, Threshold to the Origins of Agriculture. Evolutionary Anthropology. 1998;6(5):159–77. 10.1002/(sici)1520-6505(1998)6:5<159::aid-evan4>3.0.co;2-7

[3] Barlow KR, Heck M. More on Acorn Eating During the Natufian: Expected Patterning in Diet and the Archaeological Record of Subsistence In: Mason SLR, Hather J, editors. Hunter-Gatherer Archaeobotany: Perspectives from the Northern Temperate Zone. London: Institute of Archaeology, University College London; 2002 p. 128–45.

[4] Belfer-Cohen A. The Natufian in the Levant Annual Review of Anthropology. 1991;Annual Review of Anthropology (20):167–86.

[5] Boyd B. On 'Sedentism' in the Later Epipalaeolithic (Natufian) Levant. World Archaeology. 2006;38(2):164–78. 10.1080/00438240600688398

[6] Byrd BF, Colledge S. Early Natufian occupations along the edge of the southern Jordanian Steppe In: Bar-Yosef O, Valla FR, editors. The Natufian Culture in the Levant. Ann Arbor: International Monographs in Prehistory; 1991 p. 265–76.

[7] Hayden B. Sociopolitical organization in the Natufian: a view from the Northwest In: Delage C, editor. The last hunter-gatherer societies in the Near East. Oxford: Archaeopress; 2004 p. 263–308.

[8] Henry DO. Foraging, Sedentism, and Adaptive Vigor in the Natufian: Rethinking the Linkages In: Clark GA, editor. Perspectives on the Past Theoretical Biases in Mediterraneans Hunter-Gatherer Research. Philadelphia: University of Pennsylvania Press; 1991 p. 353–70.

[9] Olszewski DI. Plant Food Subsistence Issues and Scientific Inquiry in the Natufian In: Delage C, editor. The Last Hunter-Gatherers in the Near East. Oxford: British Archaeological Reports International Series S1320; 2004 p. 189–209.

[10] Rosen A. Natufian Plant Exploitation: Managing Risk and Stability in an Environment of Change. Eurasian Prehistory. 2010;7(1):113–27.

[11] Rosen A. Change and Stability in an Uncertain Environment: Foraging Strategies in the Levant form the Early Natufian through the Beginning of the Pre-Pottery Neolithic B In: Miller NF, Moore KM, Ryan K, editors. Sustainable Lifeways: Cultural Persistence in an Ever-changing Environment. Philadephia, PA: the University of Pennsylvania Press; 2011 p. 128–49.

[12] Rosen A. Shifting Household Economics of Plant Use from the Early to Late Natufian Periods of the Southern Levant In: Parker Foster, editors. New Perspectives on Household Archaeology: Eisenbrauns; 2012 p. 165–82.

[13] Rosen A. Natufian Foragers and the 'Monocot Revolution': A Phytolith Perspective In: Bar-Yosef O, Valla FR, editors. The Natufian Culture in the Levant II. Ann Arbor: University of Michigan Press; 2013 p. 638–48.

[14] Sellars JR. The Natufian of Jordan In: Henry DO, editor. The Prehistoric Archaeology of Jordan. Oxford: Archaeopress; 1998 p. 83–101.

[15] Twiss KC. Natufian Foodways: Perspectives and Potential In: Delage C, editor. The Last Hunter-Gatherers in the Near East. Oxford: Archaeopress; 2004 p. 211–28.

[16] Valla FR. The First Settled Societies—Natufian (12,500–10,200 BP) In: Levy TE, editor. The Archaeology of Society in the Holy Land. London: Leicester University Press; 1995 p. 169–89.

[17] Weinstein-Evron M. Archaeology in the Archives, Unveiling the Natufian Culture of Mount Carmel. Boston: BRILL; 2009.

[18] Valla FR, editor. Les Fouilles de la Terrasse d'Hayonim, Israël 1980–1981 et 1985–1989. Paris: De Boccard; 2012.

[19] Edwards PC. Wadi Hammeh 27, an Early Natufian Settlement at Pella in Jordan. Boston: BRILL; 2013.

[20] Rosen A. Phytolith Remains from Final Natufian Contexts at Mallaha/Eynan. Journal of the Israel Prehistoric Society. 2007;37:340–55.

[21] Maher LA, Richter T, Stock JT. The Pre-Natufian Epipaleolithic: Long-Term Behavioral Trends in the Levant. Evolutionary Anthropology. 2012;21:69–81. 10.1002/evan.21307 22499441

[22] Goring-Morris N, Belfer-Cohen A. Neolithiization processes in the Levant: the outer envelope. Current Anthropology. 2012;52:S195–S208. 10.1086/658860

[23] Richter T, Maher LA. Terminology, process and change: reflections on the Epipalaeolithic of South-west Asia. Levant. 2013;45(2):121–32. 10.1179/0075891413z.00000000020

[24] Watkins T. From foragers to complex societies in Southwest Asia In: Scarre C, editor. The Human Past: World Prehistory & the Development of Human Societies. London: Thames & Hudson; 2005 p. 201–34.

[25] Watkins T. New light on Neolithic revolution in South-west Asia. Antiquity. 2010;84(325):621–34. 10.1017/s0003598x00100122

[26] Richter T, Maher LA, Garrard A, Edinborough K, Jones MD, Stock JT. Epipalaeolithic Settlement Dynamics in southwest Asia: New Radiocarbon Evidence from the Azraq. Journal of Quaternary Science. 2013;28(5):467–79. 10.1002/jqs.2629

[27] Asouti E, Fuller D. From foraging to farming in the southern Levant: The development of Epipalaeolithic and Pre-Pottery Neolithic plant management strategies. Vegatation History and Archaeobotany. 2012;21:149–62. 10.1007/s00334-011-0332-0

[28] Snir A, Nadel D, Grosman-Yaroslavski I, Melamed Y, Sternberg M, Bar-Yosef O, et al The Origin of Cultivation and Proto-Weeds, Long Before Neolithic Farming. PLoS ONE. 2015;10(7):e0121422 10.1371/journal.pone.31422 26200895 PMC4511808

[29] Weiss E, Wetterstrom W, Nadel D, Bar-Yosef O. The broad spectrum revisited: evidence from plant remains. PNAS. 2004;101:551–5. 10.1073/pnas.0402362101 PMC470712 15210984

[30] Weiss E, Kislev ME, Simchoni O, Nadel D. Small-Grained Wild Grasses as a Staple Food at the 23 000-Year-Old Site of Ohalo II, Israel. Economic Botany. 2004;58(supplement):S125–S34. 10.1663/0013-0001(2004)58[s125:swgasf2.0.co;2

[31] Flannery KV. Origins and ecological effects of early domestication in Iran and the Near East In: Ucko PJ, Dimblebey GW, editors. The Domestication and Exploitation of Plants and Animals. Chicago: Aldine; 1969 p. 73–100.

[32] Flannery K. The Origins of Agriculture. Annual Review of Anthropology. 1973;2:271–310.

[33] Mellaart J. The Neolithic of the Near East. New York: Charles Scribner's Sons; 1975.

[34] Perrot J. La préhistoire palestiniènne. Supplement au dictionnaire de la Bible 8 Paris: Letougey & Ane; 1968 p. 286–446.

[35] Jones M, Maher LA, Macdonald D, Ryan C, Rambeau C, Richter T. The Environmental Setting of Epipalaeolithic Kharaneh IV. Quaternary International. 2016;396:95–104. 10.1016/j.quaint.2015.08.092

[36] Ramsey MN, Rosen A, Macdonald D, Maher LA, Nadel D. Sheltered by Reeds and Settled on Sedges: Construction and Use of a Twenty Thousand Year-Old Hut According to Phytolith analysis from Kharaneh IV, Jordan. in prep.

[37] Ramsey MN, Rosen A, Nadel D. Centered on the Wetlands. in prep.

[38] Ramsey MN, Rosen A. Wedded to Wetlands: Exploring Late Pleistocene Plant-Use in the Eastern Levant. Quaternary International. 2016;396:5–19. 10.1016/j.quaint.2015.10.109

[39] Rosen A. Phytolith Evidence for Environment and Plant Exploitation at Hayonim Terrace In: Valla FR, editor. Les Fouilles de la Terrasse D'Hayonim (Israel) 1980–1981 et 1985–1989. Paris: De Boccard; 2012 p. 85–92.

[40] Savard M, Nesbitt M, Jones MK. The role of wild grasses in subsistence and sedentism: new evidence from the northern Fertile Crescent. World Archaeology. 2006;38:179–96. 10.1080/00438240600689016

[41] Bettinger RL. Hunter-Gatherers Archaeological and Evolutionary Theory. New York: Plenum Press; 1991.

[42] Jochim MA. Hunter-Gatherer Subsistence and Settlement a Predictive Model New York: Academic Press; 1976.

[43] Simms SR. Behavioral Ecology and Hunter-Gatherer Foraging An example from the Great Basin. Great Britain: BAR International Series 381; 1987.

[44] Jones M, Richter T. Paleoclimatic and archaeological implications of Pleistocene and Holocene environments in Azraq, Jordan. Quaternary Research. 2011;76:363–72. 10.1016/j.yqres.2011.07.005

[45] Al-Kharabsheh A. Ground-water modeling and long-term management of the Azraq basin as an example of arid area conditions (Jodan). Journal of Arid Environments. 2000;44:143–53. 10.1006/jare.1999.0580

[46] Nobel P. Quantification of recharge to the Azraq Basin In: Dutton RW, Clarke JI, Battikhi AM, editors. Arid Land Resources and their Management: Jordan's Desert Margin. London: Kegan Paul International; 1998 p. 103–9.

[47] Macumber PG. Evolving landscapes and environment in Jordan In: MacDonald B, Adams R, Bienkowski P, editors. The Archaeology of Jordan. Sheffield: Sheffield Academic Press; 2001 p. 1–30.

[48] Garrard A, Baird D, Colledge S, Martin L, Wright K. Prehistoric Environment and Settlement in the Azraq Basin: an Interim Report on the 1987 and 1988 Excavation Seasons. Levant. 1994;26:73–109. 10.1179/lev.1994.26.1.73

[49] Garrard A, Betts A, Byrd B, Hunt C. Prehistoric Environment and Settlement in the Azraq Basin: An Interim Report on the 1985 Excavation Season. Levant. 1987;19:5–25. 10.1179/007589187790212112

[50] Garrard A, Byrd B. Beyond the Fertile Crescent: Late Palaeolithic and Neolithic Communities of the Jordanian Steppe: Oxbow Books; 2013.

[51] Montague R, Hunt G, Colledge S, Garrard A. Environment and subsistence during the Late Pleistocene and Early Holocene in the Azraq Basin. Paléorient. 1988;14(2):40–9. 10.3406/paleo.1988.4453

[52] Garrard A, Byrd B, Harvey P, Hivernel F. Prehistoric environment and settlement in the Azraq Basin. A report on the 1982 season. Levant. 1985;17:1–28. 10.1179/007589185790212105

[53] Besançon J, Geyer G, Sanlaville P. Contributions to the study of geomorphology of the Azraq Basin, Jordan In: Copeland L, Hours F, editors. The Hammer on the Rock Studies in the Early Palaeolithic of Azraq, Jordan. BAR International Series 540 Oxford: British Archaeological Reports; 1989 p. 7–63.

[54] Hillman GC, Madeyska E, Hather J. Wild plant foods and diet at Late Palaeolithic Wadi Kubbaniya (Upper Egypt): evidence from charred remains In: Wendorf F, Schild R, Close A, editors. The prehistory of Wadi Kubbaniya, vol 2: Stratigraphy, subsistence and environment. Dallas: Southern Methodist University; 1989 p. 162–242.

[55] Copeland L, Hours F. The Hammer on the Rock: Studies in the Early Palaeolithic of Azraq, Jordan. Lyon, France: Mainson de L'Orient Méditerranéen C.N.R.S.-Université Lumiére-Lyon 2; 1989.

[56] Cordova C, Nowell A, Bisson M, Ames C, Pokines J, Chang M, et al Interplacial and glacial desert refugia and the Middle Paleolithic of the Azrac Oasis, Jordan. Quaternary International. 2013;300:94–110. 10.1016/j.quaint.2012.09.019

[57] Rollefson G, Schnurrenberger D, Quintero L, al. e. Ain Soda and 'Ayn Qasiya: New Late Pleistocene and early Holocene sites in the Azraq Shishan area, eastern Jordan In: Gebel HG, Kefafi Z, Rollefson G, editors. The Prehistory of Jordan II: Perspectives from 1997. Berlin: Ex Oriente; 1997 p. 45–58.

[58] Rollefson G. Two seasons of excavation at Ain el-Assad, eastern Jordan, 1980–1981. Bulletin of the American Schools of Oriental Rsearch. 1983;252:25–34.

[59] Roskin J, Katra I, Agha N, Goring-Morris N, Porat N, Barzilai O. Rapid anthropogenic response to short-term aeolian-fluvial palaeoevironmental changes during the Late Pleistocene-Holocene transition in the northern Negev Desert, Israel. Quaternary Science Reviews. 2014;99:176–92. 10.1016/j.quascirev.2014.06.018

[60] Maher LA, Macdonald D, Allentuck A, Martin L, Spyrou A, Jones M. Occupying wide open spaces? Late Pleistocene hunter-gatherer activities in the Eastern Levant. Quaternary International. 2015;396:79–94. 10.1016/j.quaint.2015.07.054

[61] Martin L, Edwards Y, Garrard A. Hunting Practices at an Eastern Jordanian Epipalaeolithic Aggregation Site: The Case of Kharaneh IV. Levant. 2010;42(2):107–35. 10.1179/175638010x12797237885613

[62] Garrard A, Byrd B. New Dimensions to the Epipalaeolithic of the Wadi el-Jilat in central Jordan. Paléorient. 1992;18:47–62. 10.3406/paleo.1992.4562

[63] Maher LA, Richter T, Macdonald D, Jones MD, Martin L, Stock JT. Twenty Thousand-Year-Old Huts at a Hunter-Gatherer Settlement in Eastern Jordan. PLoS ONE. 2012;7(2):e31447 10.1371/journal.pone.0031447 22355366 PMC3280235

[64] Jones J. Using gazelle dental cementum studies to explore seasonality and mobility patterns of the Early-Middle Epipalaeolithic Azraq Basin, Jordan. Quaternary International. 2012;252:195–201. 10.1016/j.quaint.2011.09.001

[65] Richter T, Garrard A, Allcock S, Maher LA. Interaction before Agriculture: exchanging material and sharing knowledge in the Final Pleistocene Levant. Cambridge Archaeological Journal. 2011;21:95–114. 10.1017/s0959774311000060

[66] Maher LA, Macdonald D. Exploring Typo-technological Diversity in Chipped Stone from Epipaleolithic Kharaneh IV, Eastern Jordan. CBRL Bulletin. 2012;7:42–5.

[67] Richter T, Garrard A, Allock S, Maher LA. Interaction before Agriculture: Exchanging Material and Sharing Knowledge in the Final Pleistocene Levant. Cambridge Archaeological Journal. 2011;21(1):95–114. 10.1017/s0959774311000060

[68] Muheisen M. The Epipalaeolithic phases of Kharaneh IV In: Garrard A, Gebel HG, editors. The Prehistory of Jordan The State of Research in 1986. 396 (i). Great Britain: B.A.R. Publications; 1988 p. 353–67.

[69] Maher LA, Richter T, Jones M, Stock JT. 2009 Excavations at the Epipalaeolithic Site of Kharaneh IV. Report to the Jordanian Department of Antiquities: manuscript in the posession of the author; 2009.

[70] Maher LA, Richter T, Stock JT. 2008 Excavations at the Epipalaeolithic Site of Kharaneh IV. Report for the Jordanian Department of Antiquities: manuscript in the posession of the author; 2008.

[71] Maher LA, Richter T, Stock JT. 2010 Excavations at the Epipalaeolithic Site of Kharaneh IV. Report to the Jordanian Department of Antiquities: manuscript in the posession of the author; 2010.

[72] Maher LA, Macdonald D. 2013 Excavations at the Epipalaeolithic Site of Kharaneh IV. report to the Jordanian Department of Antiquities: manuscript in the posession of the author; 2013.

[73] Zeder MA. The Broad Spectrum Revolution at 40: Resource diversity, intensification, and an alternative to optimal foraging explanations. Journal of Anthropological Archaeology. 2012;31(3):241–64. 10.1016/j.jaa.2012.03.003

[74] Simon HA. Rational Choice and the Structure of the Environment. Psychological Review. 1956;63(2):129–38. 10.1037/h0042769 13310708

[75] Butzer K. Archaeology as human ecology Cambridge: Cambridge University Press; 1982.

[76] Rosen A, Rivera-Collazo I. Climate change, adaptive cycles, and the persistence of foraging economies during the late Pleistocene/Holocene transition in the Levant. PNAS. 2012;109(10):3640–5. 10.1073/pnas.1113931109 22371591 PMC3309764

[77] Byrd BF, Garrard A, Brandy P. Modeling foraging ranges and spatial organization of Late Pleistocene hunter-gatherers in the southern Levant—A least-cost GIS approach. Quanternary International. 2016;396:62–78. doi: http://dx/doi.org/10.1016.j.guaint.2015.07.048

[78] Wrangham R, Cheney D, Seyfarth R, Sarmiento E. Shallow-Water Habitats as Sources of Fallback Foods for Hominins. American Journal of Physical Anthropology. 2009;140:630–42. 10.1002/ajpa.21122 19890871

[79] Childe VG. New Light on the Most Ancient East. London: Routledge & Kegan Paul Limited; 1952.

[80] Keddy PA. Wetland Ecology Principles and Conservation. Cambridge: Cambridge University Press; 2000.

[81] Ramsey MN, Jones M, Richter T, Rosen A. Modifying the Marsh: A Preliminary Evaluation of Early Epipaleolithic Hunter-Gatherer Impacts in the Azraq Wetland. The Holocene. 2015;25:1553–64. 10.1177/0959683615594240

[82] Bettinger RL, Wohlgemuth E. Archaeological and Ethnographic Evidence for Indigenous Plant Use in California In: Smith BD, editor. The Subsistence Economies of Indigenou North American Societies A Handbook. Washington, D.C.: Smithsonian Institution Scholarly Press; 2011 p. 113–30.

[83] Gibson DJ. Grasses and Grassland Ecology. Oxford: Oxford University Press; 2009.

[84] Lee RB. What Hunters Do for a Living, or, How to Make Out on Scarce Resources In: Lee RB, DeVore I, editors. Man the Hunter. Chicago: Aldine Publishing Company; 1968 p. 30–48.

[85] Binford LR. Willow Smoke and Dogs' Tails: Hunter-Gatherer Settlement Systems and Archaeological Site Formation. American Antiquity. 1980;45(1):4–20. 10.2307/279653

[86] Rosen A. Phytolith Protocol. Manuscript in posession of author1999.

[87] Rosen A. Phytolith analysis in Near Eastern archaeology In: Pike S, Gitin S, editors. The Practical Impact of Science on Near Eastern and Aegean Archaeology. Weiner Laboratory monograph 3 London: Archaetype Publications; 1999 p. 9–15.

[88] Albert RM, Lavi O, Estroff S, Weiner S, Tsatskin A, Ronen A, et al Modes of occupation of Tabun Cave, Mt. Carmel, Israel during the Mousterian Period: a study of sediments and phytoliths. Journal of Archaeological Science. 1999;26:1249–60. 10.1006/jasc.1999.0355

[89] Albert RM, Bar-Yosef O, Meignen L, Weiner S. Quantitative phytolith study at hearths from the Natufian and Middle Paleolithic levels of Hayonim Cave (Galilee, Israel). Journal of Archaeological Science. 2003;30:461–80. 10.1006/jasc.2002.0854

[90] Power RC, Rosen A, Nadel D. The Economic and Ritual Utilization of Plants at the Raqefet Cave Natufian site: The evidence from phytoliths. Journal of Anthropological Archaeology. 2014;33:49–65. 10.1016/j.jaa.2013.11.002

[91] Madella M, Jones M, Echlin MK, Powers-Jones AH, Moore M. Plant water availability and analytical microscopy of phytoliths: implications for ancient irrigation in arid zones. Quanternary International. 2009;193:32–40. 10.1016/j.quaint.2007.06.012

[92] Liu L, Jie D, Liu H, Li N, Guo J. Response of phytoliths in *Phragmites communis* to humidity in NE China. Quanternary International. 2013;304:193–9. 10.1016/j.quaint.2013.03.020

[93] Albert RM, Weiner S, Bar-Yosef O, Meignen L. Phytoliths in the Middle Palaeolithic Deposits of Kebara Cave, Mt Carmel, Israel: Study of the Plant Materials used for Fuel and Other Purposes. Journal of Archaeological Science. 2000;27:931–47. 10.1006/jasc.2000.0507

[94] Albert RM, Weiner S. Study of phytoliths in prehistoric ash layers using a quantitative approach In: Meunier J-D, Colin F, editors. Phytoliths: Applications in Earth Sciences and Human History. Netherlands: A.A. Balkema Publishers; 2001 p. 251–66.

[95] Madella M, Alexandre A, Ball T. International code for phytolith nomenclature. Annals of Botany. 2005;96(2):253–60. 10.1093/aob/mci172 15944178 PMC4246872

[96] Metcalf C. Anatomy of the monocotyledons I. Gramineae. London: Oxford University Press; 1960.

[97] Twiss PC. Predicted World DIstribution of C3 and C4 Grass Phytoliths In: Rapp GJ, Mulholland SC, editors. Phytolith Systematics Emerging Issues. New York: Plenum Press; 1992 p. 113–28.

[98] Novello A, Barboni D. Grass inflorescence phytoliths of useful species and wild cereals from sub-Saharan Africa. Journal of Archaeological Science. 2015;59:10–22. 10.1016/j.jas.2015.03.031

[99] Rosen A. Preliminary identfication of silica skeletons from Near Eastern archaeological sites: an anatomical approach In: Rapp GJ, Mulholland SC, editors. Phytolith Systematics, Emerging Issues. Advances in Archaeological and Museum Science. New York: Plenum Press; 1992 p. 129–47.

[100] Twiss PC, Suess E, Smoth RM. Morphological Classification of Grass Phytoliths. Soil Science Society of America Proceedings. 1969;33(1):109–16. 10.2136/sssaj1969.03615995003300010030x

[101] Andrejko MJ, Cohen AD. Scanning electron microscopy of silicophytoliths from the Okefenokee swamp-marsh complex In: Cohen AD, Casagrande DJ, Andrejko MJ, Best GR, editors. The Okefenokee swamp: its natural history, geology and geochemistry. Wetland Surveys, Los Alamos, NM 1984 p. 468–91.

[102] Bremond L, Alexandre A, Peyron O, Guiot J. Grass water stress estimated from phytoliths in West Africa. Journal of Biogeography. 2005;32:311–27. 10.1111/j.1365-2699.2004.01162.x

[103] Sangster AG, Parry DW. Some Factors in Relation to Bulliform Cell Silicification in the Grass Leaf. Annals of Botany. 1969;33:315–23.

[104] Ryan P. Plants as material culture in the Near Eastern Neolithic: Perspectives from the silica skeleton artifactual remains at Çatalhöyük. Journal of Anthropological Archaeology. 2011;30(3):292–305. 10.1016/j.jaa.2011.06.002

[105] Ryan P. Diversity of Plant and Land Use During the Near Eastern Neolithic: Phytolith Perspectives from Çatalhöyük [unpublished doctoral thesis]: University College London; 2009.

[106] Greiss EAM. Anatomical Identification of some Ancient Egyptian Plant Materials Le Claire, Impremerie Costa Tsoumas & Co.; 1957.

[107] Ollendorf A, L., Mulholland SC, Rapp GJ. Phytoliths from some Israeli Sedges. Israel Journal of Botany. 1987;68:125–32.

[108] Ollendorf A L. Towards a Classification Scheme of Sedge (Cyperaceae) Phytoliths In: Rapp GJ, Mulholland SC, editors. Phytolith Systematics Emerging Issues. New York Plenum Press; 1992 p. 91–112.

[109] Metcalf C. Anatomy of the Monocotyledons V. Cyperaceae. London: Oxford University Press; 1971.

[110] Le Cohu M-C. Examen au microscope électronique à balayage, des cônes de silice chez les Cypéracées. C R Acad Sc Paris. 1973;277:1301–3.

[111] Bozarth SR. Biosilicate assemblages of boreal forests and aspend parklands In: Pearsall DM, Piperno DR, editors. Current research in phytolith analysis: Applications in archaeology and paleoecology. Pennsylvania: The University Museum of Archaeology and Anthropology, University of Pennsylvania; 1993 p. 95–101.

[112] Bozarth SR. Classification of Opal Phytoliths Forms in Selected Dicotyledons Natives to the Great Plains In: Rapp GJ, Mulholland SC, editors. Phytolith Systematics Emerging Issues. New York: Plenum Press; 1992 p. 193–214.

[113] Twiss PC. A Curmudgeon's view of grass phytolithology In: Meunier J-D, Colin F, editors. Phytoliths Applications in Earth Science and Human History. Lisse: Swets & Zeitlinger B.V.; 2001 p. 7–26.

[114] Caracuta V, Weinstein-Evron M, Yeshurun R, Kaufman D, Tsatskin A, Boaretto E. Charred wood remains in the natufian sequence of el-Wad terrace (Israel): New insights into the climatic, environmental and cultural changes at the end of the Pleistocene. Quaternary Science Reviews. 2016;131:20–32. 10.1016/j.quascirev.2015.10.034

[115] Steward JH. Basin-Plateau aboriginal sociopolitical groups. Bur Am Ethnol Bull. 1938;116:1–346.

[116] Steward JH. Ethnography of the Owens Valley Paiute In: Kroeber AL, Lowie RH, Olson RL, editors. University of California Publications in American Archaeology and Ethnology. XXXIII Berkeley, California: University of California Press; 1934 p. 233–350.

[117] Fowler CS, Rhode D. Plant Foods and Foodways among the Great Basin's Indigenous Peoples In: Smith BD, editor. The Subsistence Economies of Indigenous North American Societies A Handbook. Washington, D.C.: Smithsonian Institution Scholarly Press; 2011 p. 233–70.

[118] Fowler CS. Ethnographic Perspectives on Marsh-Based Cultures in Western Nevada. In: Janetski JC, Madsen DB, editors. Wetland Adaptations in the Great Basin. Occasional Papers No. 1. Provo, Utah: Brigham Young University 1990. p. 17–32.

[119] Fowler CS. Tule Technology Northern Pauite Uses of Marsh Resources in Western Nevada. Washington, D.C.: Smithsonian Institution Press; 1990.

[120] Fowler CS. Food-Named Groups Among Northern Paiute in North America's Great Basin: An Ecological Interpretation In: Williams NM, Hunn ES, editors. Resource Managers: North American and Australian Hunter-Gatherers. Boulder, Colorado: Westview Press, Inc.; 1982 p. 113–30.

[121] Ebeling W. Handbook of Indian Foods and Fibers of Arid America. Berkeley: University of California Press; 1986.

[122] Gott B. Cumbungi, *Typha* species, a staple Aboriginal food in southern Australia. Australian Aboriginal Studies. 1999;1:33–50.

[123] Gott B. Ecology of root use by the Aborigines of southern Australia. Archaeology in Oceania. 1982;17:59–67. 10.1002/j.1834-4453.1982.tb00039.x

[124] Wollstonecroft MM, Hrudová Z, Hillman GC, Fuller D. Bolboschoenus glaucus (Lam.) S.G. Smith, a new species in the flora of the ancient Near East. Vegetation History and Archaeobotany. 2011;20:459–70. 10.1007/s00334-011-0305-3

[125] Wollstonecroft MM, Ellis PR, Hillman GC, Fuller D. Advancements in plant food processing in the Near Eastern Epipalaeolithic and implications for improved edibility and nutrient bioaccessibility: an experimental assessment of sea club-rush (*Bolboschoenus maritimus* (L) Palla). Vegetation History and Archaeobotany. 2008;17(1):S19–S27.

[126] Wollstonecroft MM. Harvesting experiments on the clonal marophyte sea club-rush (*Bolboschoenus maritimus* (L.) Palla): an approach to identifying variables that may have influened hunter-gatherer resource selection in Late Pleistocene Southwest Asia In: Fairbairn AS, Weiss E, editors. From foragers to farmers: papers in honour of Gordon C Hillman. Oxford: Oxbow Monographs; 2009 p. 127–38.

[127] Wollstonecroft MM. Post-Harvest Intensification in Late Pleistocene Southwest Asia: Plant Food Processing as a Critical Variable in Epipaleolithic Subsistence and Subsistence Change [Unpublished PhD Thesis]: University College London; 2007.

[128] Piperno DR, Weiss E, Holst I, Nadel D. Processing of wild cereal grains in the Upper Palaeolithic revealed by starch grain analysis. Nature. 2004;430:670–3. 10.1038/nature02734 15295598

[129] Nadel D, Piperno DR, Holst I, Snir A, Weiss E. New Evidence for the processing of wild cereal grains at Ohalo II, a 23 000-year-old campsite on the shore of the Sea of Galilee, Israel. Antiquity. 2012;86:990–1003. 10.1017/s0003598x00048201

[130] Kislev ME, Nadel D, Carmi I. Epipalaeolithic (19, 000 BP) cereal and fruit diet at Ohalo II, Sea of Galilee, Israel. Review of Palaeobotany and Palynology. 1992;73:161–6. 10.1016/0034-6667(92)90054-k

[131] Weiss E, Kislev ME, Simchoni O, Nadel D, Tschauner H. Plant-food preparation area on an Upper Paleolithic brush hut floor at Ohalo II, Israel. Journal of Archaeological Science. 2008;35(2):400–14.

[132] Snir A, Nadel D, Weiss E. Plant-food preparation on two consecutive floors at Upper Paleolithic Ohalo II, Israel. Journal of Archaeological Science. 2015;53:61–71. 10.1016/j.jas.2014.09.023

[133] Rowley-Conwy P. Time, change and the archaeology of hunter-gatherers: how originial is the 'Original Affluent Society'? In: Panter-Brick C, Layton R, Rowley-Conwy P, editors. Hunter-Gatherers an Interdisciplinary Perspective. Cambridge: Cambridge University Press; 2001 p. 39–72.

